# P-15 promotes chondrocyte proliferation in osteoarthritis by regulating SFPQ to target the Akt-RUNX2 axis

**DOI:** 10.1186/s13018-023-03658-z

**Published:** 2023-03-13

**Authors:** Yuanli Li, Junlan Nie, Changgong Deng, Hong Li

**Affiliations:** 1 Affiliated Hospital of North Sichuan Medical College, 1 Maoyuan South Road, Shunqing District, Nanchong, 637000 Sichuan China; 2 Affiliated Hospital of North Sichuan Medical College, Nanchong, 637000 Sichuan China; 3 Anatomy Teaching and Research Section, Affiliated Hospital of North Sichuan Medical College, Nanchong, 637000 Sichuan China

**Keywords:** Chondrocytes, P-15 peptide, SFPQ, Akt-RUNX2 axis, Proliferation

## Abstract

**Background:**

The disruption of chondrocyte proliferation and differentiation is a critical event during the process of joint injury in osteoarthritis (OA). P-15 peptides could bind to integrin receptors on various precursor cells, promote cell adhesion, release growth factors, and promote the differentiation of osteoblast precursor cells. However, the role of P-15 in OA, particularly in chondrocyte proliferation, is not fully understood.

**Methods:**

The activity of SFPQ and RUNX2 in the bone tissue of patients with osteoarthritis was analyzed using quantitative real-time polymerase chain reaction (qRT-PCR). Interleukin-1β (IL-1β) inducer was performed to establish an in vitro model of OA. Cell proliferation was measured by CCK-8 assay. The expressions of COL2a1, ACAN, COMP, SOX9, and BMP2 related to cartilage differentiation were detected using qRT-PCR. In addition, the expression levels of SFPQ, AKT, p-AKT, and RUNX2 were detected using Western blotting.

**Results:**

The results showed that the expression of SFPQ was significantly decreased and the expression of RUNX2 was significantly increased in osteoarthritis cartilage tissue. P-15 peptide reversed IL-1β-induced cell proliferation obstruction and alleviated chondrocyte damage. Furthermore, P-15 polypeptide increased the expression levels of cartilage differentiation genes COL2a1, ACAN, and BMP2, while decreasing the expression of COMP and SOX9 in an inverse dose-dependent manner. Then specific interfering RNA proved that P-15 maintains chondrocyte stability and is associated with the SFPQ gene. Finally, we confirmed that P-15 inhibited the Akt-RUNX2 pathway, which is regulated in the expression of SFPQ.

**Conclusions:**

P-15 can mitigate chondrocyte damage and osteoarthritis progression by inhibiting cell death and modulating SFPQ-Akt-RUNX2 pathway, offering an opportunity to develop new strategies for the treatment of osteoarthritis.

## Introduction

Osteoarthritis (OA) is a chronic joint disease characterized by degenerative articular cartilage changes and secondary hyperosteogeny [1], affecting 10–15% of adults over the age of 60 worldwide [2]. OA is a major cause of disability, and its incidence, which is related to sex, age, obesity, joint trauma, and other factors, is on the rise, bringing a huge economic burden to patients and society [3, 4]. The pathogenesis of OA involves a redox state imbalance leading to oxidative stress, aging, and apoptosis in chondrocytes, as well as decreased anabolism and increased catabolism of the extracellular matrix [5, 6]. After articular cartilage injury, it is mainly repaired by the proliferation and differentiation of chondrocytes. However, the proliferative capacity of chondrocytes is limited, and the quality and quantity of chondrocytes directly affect the repair [7]. Therefore, regulating chondrocyte proliferation and elucidating its molecular mechanism are of great significance for developing effective therapeutic strategies.

SFPQ is a key nuclear protein regulator (Splicing Factor Proline and Glutamine Rich), which is overexpressed in tumors and functions to promote tumor cells proliferation, chemical resistance, and invasion [8, 9]. Runt-associated transcription factor 2 (RUNX2) is a master transcription factor for chondrocyte hypertrophy that plays an important role in osteoarthritis [10]. Researchers have demonstrated that chondrocyte hypertrophic differentiation, a key process in endochondral ossification, is also a feature of osteoarthritis leading to cartilage destruction [10, 11]. In addition, SFPQ could be competitively bound by various non-coding RNAs, thereby increasing the translation level of RUNX2 [12, 13]. High expression of RUNX2 leads to chondrocyte hypertrophy and cartilage extracellular matrix decomposition, promoting cartilage degeneration in knee osteoarthritis cartilage [14].

Akt (also known as protein kinase B) is a serine/threonine protein kinase directly activated by PI3K [15]. The PI3K/Akt pathway can be activated in response to insulin, growth factors, and cytokines, thereby regulating a wide range of processes such as glucose metabolism, biosynthesis, and redox balance [16, 17]. Currently, findings have reported the osteo-inductive potential of a heparinmediated Wnt3a-phosphoinositide 3-kinase/Akt-RUNX2 signaling network [18]. Furthermore, activation of the PI3K-Akt-RUNX2 pathway and its downstream effectors can enhance osteoblast activity and stimulate osteogenesis [19].

Collagen comprises > 90% of the spatially fixed matrix of bone and is a major regulator of cell adhesion [20] and osteogenic differentiation [21]. Bhatnagar et al. [22] have identified a potent cell-binding domain of human Type-I collagen in the a1(I) chain sequence (P-15) 766GTPGPQGIAGQRGVV780. Previous studies have shown that anorganic bone mineral (ABM) particles coated with P-15 peptides can mimic the bone matrix components and facilitate bone regeneration [23, 24]. To clarify the relationship between P-15 regulation of chondrocyte proliferation and SFPQ-Akt-RUNX2 pathway, we chemically detected the expression of SFPQ and RUNX2 in the bone joint samples from clinical osteoarthritis and non-osteoarthritis patients. Afterward, mouse osteoarticular chondrocytes were co-cultured with P-15 peptide preparations and treated with IL-1β to establish a cellular inflammation model. The effect and mechanism of P-15 on OA chondrocytes were evaluated for cell proliferation activity, paranuclear plaque formation, and Akt-RUNX2 pathway regulation.

## Materials and methods

### Human osteoarthritis sample collection

Human normal samples were collected from amputees. Articular cartilage samples from 5 successive patients with knee OA were obtained at the time of total knee arthroplasty. All participants have signed the informed patient consent, and the protocols for collecting and analyzing human articular cartilage were approved by the Ethical Committee of Affiliated Hospital of North Sichuan Medical College. All experimental procedures followed the ethical standards of the responsible committee on human experimentation (institutional and national) and with the Declaration of Helsinki [25].

### Chondrocyte culture

Primary chondrocytes were derived from cartilage specimens obtained from mice (within 24 h). Briefly, for extraction, the cartilage blocks were first cleaned, sheared and digested with 0.2% collagenase II (Sigma, USA) at 37 ℃ and then the cell pellets were harvested for culture. The culture medium for chondrocytes was DMEM (Dulbecco’s modified Eagle medium) supplemented with 10% fetal bovine serum (Gibco, USA) and cultured in culturing dishes at 37℃ and 5% CO_2_. After 48 h, the liquid was changed once every 2–3 days, and growth and morphology of the cells were observed under an inverted microscope.

### Cell treatment and grouping

First, P-15 peptides were plated on the surface of cell culture wells at different concentrations, and the cells were treated with 10 mg·L^−1^ IL-1β (Sigma, USA) for 24 h to construct an in vitro model of OA when the cells in the cell culture plate were confluent to 60–80%. Simultaneously, mouse primary chondrocytes without any treatment were used as a control, and IL-1β-treated cells without P-15 were used as OA group. Finally, after 24–72 h of co-cultivation, cell samples were collected for subsequent detection.

### siRNA transfection

The optimal concentration of P-15 was used in subsequent experiments. According the SFPQ mRNA sequence published on NCBI (NM_005066), the siRNA design software (BLOCK-iTTM RNAi Designer) of the Invitrogen website was used to design interference primers (Table 1). Cells were transfected with Lipofectamine™ 2000 (CAT. No. 11668–019, Invitrogen) under instructions. Lipofectamine™ 2000 and siRNA at the recommended dose were added into the medium, mixed the solution and let stand for 20 min. Lip2000-siRNA mixed solution treated cells for 6 h and then replaced with 2% FBS maintenance medium for another 36 h.

**Table 1:** siRNA sequence design targeting SFPQ gene

Sequence name		Sequence of siRNA
siRNA SFPQ	Sense	5’-CAUGGCACGUUUGAGUACGAAUAUU-3’
	Anti-sense	5’-AAUAUUCGUACUCAAACGUGCCAUG-3’
siRNA negative control	Sense	5’-UUCUCCGAACGUGUCACGUTT-3’
	Anti-sense	5’-ACGUGACACGUUCGGAGAATT-3’

### RNA extraction and qRT-PCR

Total RNA was extracted using a RNeasy mini kit (Qiagen, Germany) according to the manufacturer’s instructions. Extracted RNA was quantitatively evaluated using a NanoDrop spectrophotometer (NanoDrop Technologies, USA). The HiScript Reverse Transcriptase Kit (Vazyme, China) was used to convert RNA into cDNA. The setup program was to perform reverse transcription at 37 ℃ for 15 min, followed by termination of the reaction at 85 ℃ for 5 s. Finally, the DNA amplification process was performed using a Step one Plus real-time PCR system (Applied Biosystems). The required primers included β-actin, SFPQ, RUNX2, COL2a1, ACAN, COMP, SOX9, and BMP2. The β-actin was used as the reference gene for the evaluation of mRNA expression, and the ^2 ΔΔ^Cq method was used to calculate the levels of gene expression [26]. Each experiment was repeated 3 times. Primer sequences are shown in Table 2.

**Table 2:** RT-qPCR primers in this study

Name	Primer sequences
SFPQ-F	5’-TGGACAACAGAGCGAGAC-3’
SFPQ-R	5’-AACAGAAGTAGCACAAGGAGAT-3’
RUNX2-F	5’-CGTCCACTGTCACTT TAATAGCTC-3’
RUNX2-R	5’-GTAGCCAGGTTCAACGATCTG-3’
COL2a1-F	5’-CTACGGTGTCAGGGCCAG-3’
COL2a1-R	5’-GTGTCACACACACAGATGCG-3’
ACAN-F	5’-CAGGCTATGAGCAGTGTGATGC-3’
ACAN-R	5’-GCTGCTGTCTTTGTCACCCACA-3’
COMP-F	5’-ACTGCCTGCGTTCTAGTGC-3’
COMP-R	5’-CGCCGCATTAGTCTCCTGAA-3’
SOX9-F	5’-CGGCTCCAGCAAGAACAA-3’
SOX9-R	5’-TGCGCCCACACCATG-3’
BMP2-F	5’-TGGACGCTCTTTCAATGGAC-3’
BMP2-R	5’-AGCAGCAACGCTAGAAGAC-3’
β-actin-F	5’-CGTCCCGTAGACAAAATGGT-3’
β-actin-R	5’-TTGATGGCAACAATCTCCAC-3’

### Cell viability assay

In order to assess the effect of P-15 on chondrocyte activity, the cell counting kit-8 (CCK-8) assay (Dojindo Co., Kumamoto, Japan) was used according to manufacturer’s protocol. The same density of chondrocytes was seeded in a 96-well plate that treated with P-15 and cultured for 24, 48, and 72 h, respectively. After washed with PBS, the cells in each well were incubated with 10% CCK-8 solution at 37℃ for 2 h. The absorbance was measured at 450 nm by a microplate reader (Thermo Scientific, Logan, UT, USA).

### Western blotting

Western blotting was performed as previously described [27]. RIPA lysis buffer with 1 mM protein phosphatase inhibitor was added into cells for protein extraction. The lysate was collected and centrifuged at 12,000 r/min for 10 min at 4 ℃. The BCA protein assay kit (Thermo Scientific) was used to determine the concentration of proteins. The proteins were separated by sodium dodecyl sulfate-polyacrylamide gel electrophoresis (SDS-PAGE) and then transferred onto PVDF membranes (Bio-Rad, Hercules, CA, USA). Membranes were blocked with 5% BSA (Sigma-Aldrich, Darmstadt, Germany)-TBST buffer solution (Bio Froxx, Guangzhou, China) for 1 h at room temperature, incubated with antibodies against SFPQ (ab38148, Abcam, U.K.), AKT (GTX121937, GeneTex, USA), p-AKT (WLP001a,Shenyang Wanlei Biotechnology Co., Ltd., China), RUNX2 (ab23981, Abcam, U.K.), and β-actin (30101ES, Shanghai Yisheng Biological Co., Ltd., China) at 4 ℃ overnight. Subsequently, incubated with horseradish peroxidase-conjugated secondary antibodies (SE134, SA131, Solarbio, China) for 1 h. Protein bands were detected using a chemiluminescence system (Bio-Rad Laboratories, Hercules, CA, USA) with an enhanced chemiluminescence (ECL) kit (Millipore, Darmstadt, Germany). Signal intensity was compared using the Image J software (NIH, Bethesda, MD, USA).

### Statistical analysis

Data were analyzed using GraphPad Prism 8.0 (GraphPad, La Jolla, CA, USA) and SPSS v26.0 (IBM Corp., USA). The two independent group samples used the *t*-test, and the multiple group samples used a one-way analysis of variance (ANOVA) followed by Tukey’s test. *P* values less than 0.05 were considered significant.

## Results

### SFPQ and RUNX2 expression are associated with OA progression

**Fig. 1:**
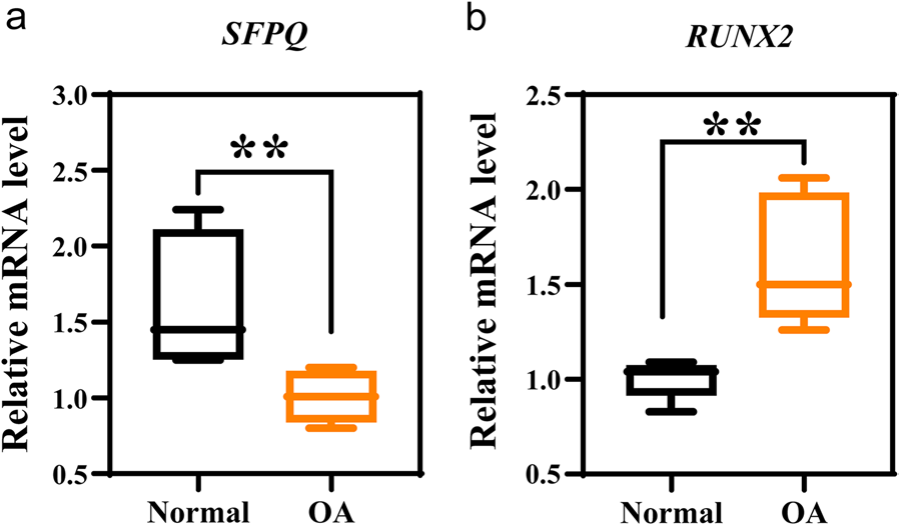
Expression patterns of SFPQ and RUNX2 in healthy people and OA patients. **a** SFPQ mRNA was detected by RT-qPCR in normal (control) and OA groups. **b** RUNX2 mRNA was detected by RT-qPCR in normal (control) and OA groups. Data are presented as mean ± SD. ^**^P < 0.01, *n* = 5

To ascertain whether SFPQ and RUNX2 play a role in OA pathogenesis, we first analyzed the expression patterns of SFPQ in human bone joint samples with normal and OA via real-time PCR (Fig. 1A). SFPQ was broadly expressed in normal bone joints, but it was significantly lower in patients with osteoarthritis (Fig. 1a). In addition, the transcript levels of RUNX2 showed the opposite trend (Fig. 1b). The changes in SFPQ and RUNX2 expression in patients with OA indicate the potential importance of SFPQ and RUNX2 in the progression of arthritis.

### P-15 peptides enhances chondrocyte activity inhibited by IL-1β

**Fig. 2:**
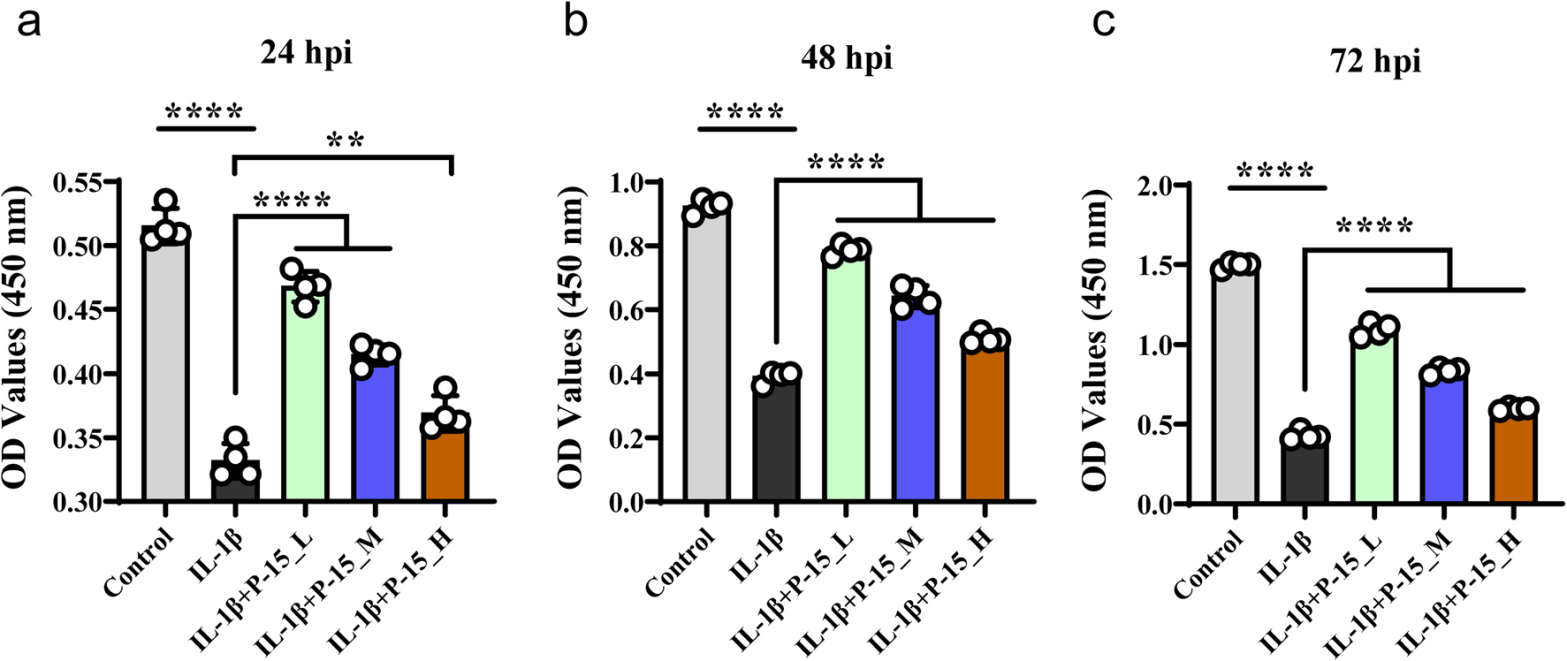
Effects of P-15 on the activity of chondrocytes. Chondrocytes were treated with P-15 (concentrations were 0.1%, 0.5% and 1% respectively) and IL-1β for 24 h **a**, 48 h **b**, or 72 h **c** according to the above method, and a CCK-8 kit was used to detect the cell activity in each group. Data are presented as mean ± SD. ^**^P < 0.01, *n* = 4

It has been reported that P-15 could enhance cell adhesion, migration, and survival [28, 29]. Thence, we investigated the potential role of P-15 in chondrocytes by co-culturing P-15 with IL-1β-induced OA cells. As shown in Fig. 2a–c, IL-1β treatment significantly suppressed the overall activity of chondrocytes, while the effects of which were rescued by P-15 peptides. Interestingly, the rescue effect of P-15 was dose-dependently diminished, scilicet, in the concentration gradient used here, the lower P-15 dose, the stronger the reversal effect on IL-1β-induced inhibition of cell activity. In addition, chondrocyte activity increased with time in each group during 24–72 hpi (Table 3).

**Table 3:** Effect of P-15 peptide on proliferation activity of IL-1β-induced chondrocytes (*χ* ± SD)

Groups	Cell activity (450 nm)			*F*	*P*
	24 hpi	48 hpi	72 hpi		
Control	0.515175 ± 0.01389	0.923625 ± 0.02226 **a**	1.496300 ± 0.02120 **a, b**	236.3	0.000
IL-1β	0.331750 ± 0.01367	0.391350 ± 0.01942 **a**	0.428000 ± 0.02833 **a, b**	116.8	0.000
IL-1β + P-15_L	0.467750 ± 0.01197	0.786100 ± 0.01709 **a**	1.091250 ± 0.04127 **a, b**	213.0	0.000
IL-1β + P-15_M	0.414675 ± 0.00792	0.641000 ± 0.03437 **a**	0.835100 ± 0.02137 **a, b**	175.1	0.000
IL-1β + P-15_H	0.368775 ± 0.01398	0.509200 ± 0.01550 **a**	0.597325 ± 0.01113** a, b**	146.9	0.000

Compared with 24 hpi, **a**, *P* < 0.05; compared with 48 hpi, **b**, *P* < 0.05

### P-15 regulates SFPQ to promote paranuclear plaque formation and chondrogenesis

**Fig. 3:**
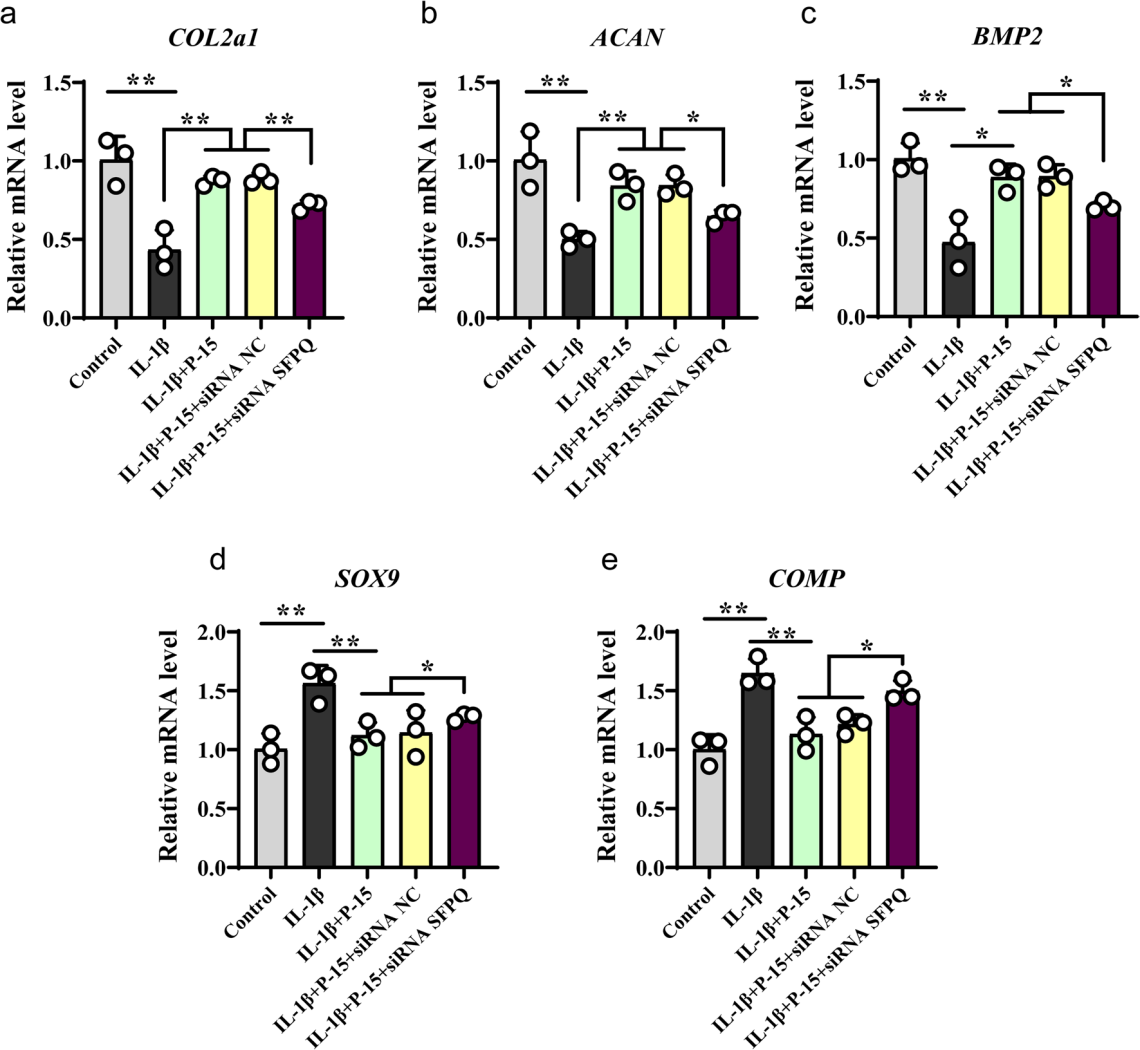
Effects of SFPQ expression on cartilage markers in cell co-culture with P-15. Cells were treated as previously described and the expression levels of chondrogenesis markers COL2a1 **a**, ACAN **b**, BMP2 **c**, SOX9 **d** and COMP **e** was detected by RT-qPCR. COL2a1, ACAN, BMP2 increased and SOX9, COMP reduced compared with the IL-1β-induced group during co-cultivation when SFPQ gene expression decreased. Data are presented as mean ± SD. ^*^P < 0.05, ^**^P < 0.01,* n* = 3

Since we confirmed the low expression of SFPQ in osteoarthritis patients, the literature has suggested that SFPQ protein is necessary for the formation of parinuclear plaques [30]. To elucidate whether P-15 regulates SFPQ to participate in para-nuclear plaque formation and chondrogenesis, and to investigate how P-15 influence this process, we performed micromass culture. Then RT-qPCR was implemented to confirm the expression of cartilage differentiation-related indicators. In cultured mouse chondrocytes, exposure to the pro-inflammatory factor IL-1β resulted in significant down-regulation of three chondrogenic differentiation-related anabolic factors (COL2a1, ACAN, and BMP2), which could be partially reversed by co-culturing P-15 peptides (Fig. 3a–c). However, transfection of SFPQ siRNA again attenuated their expression (Fig. 3a–c), suggesting that knockdown of SFPQ favors IL-1β-induced adverse reactions. Consistently, SOX9 and COMP, which were stimulated by IL-1β, could be reversed by supplementation of P-15 peptides, which were also upregulated by SFPQ siRNA (Fig. 3d and e). These results suggested that the positive effect of P-15 in OA chondrocytes is related to the regulation of SFPQ expression. Together, these findings demonstrated that P-15 promotes the formation of parinuclear plaques by targeting SFPQ gene in the pathological process of OA, affects the proliferation of chondrocytes, and inhibits the development of OA.

### P-15 regulates Akt-RUNX2 signaling pathway through SFPQ

**Fig. 4:**
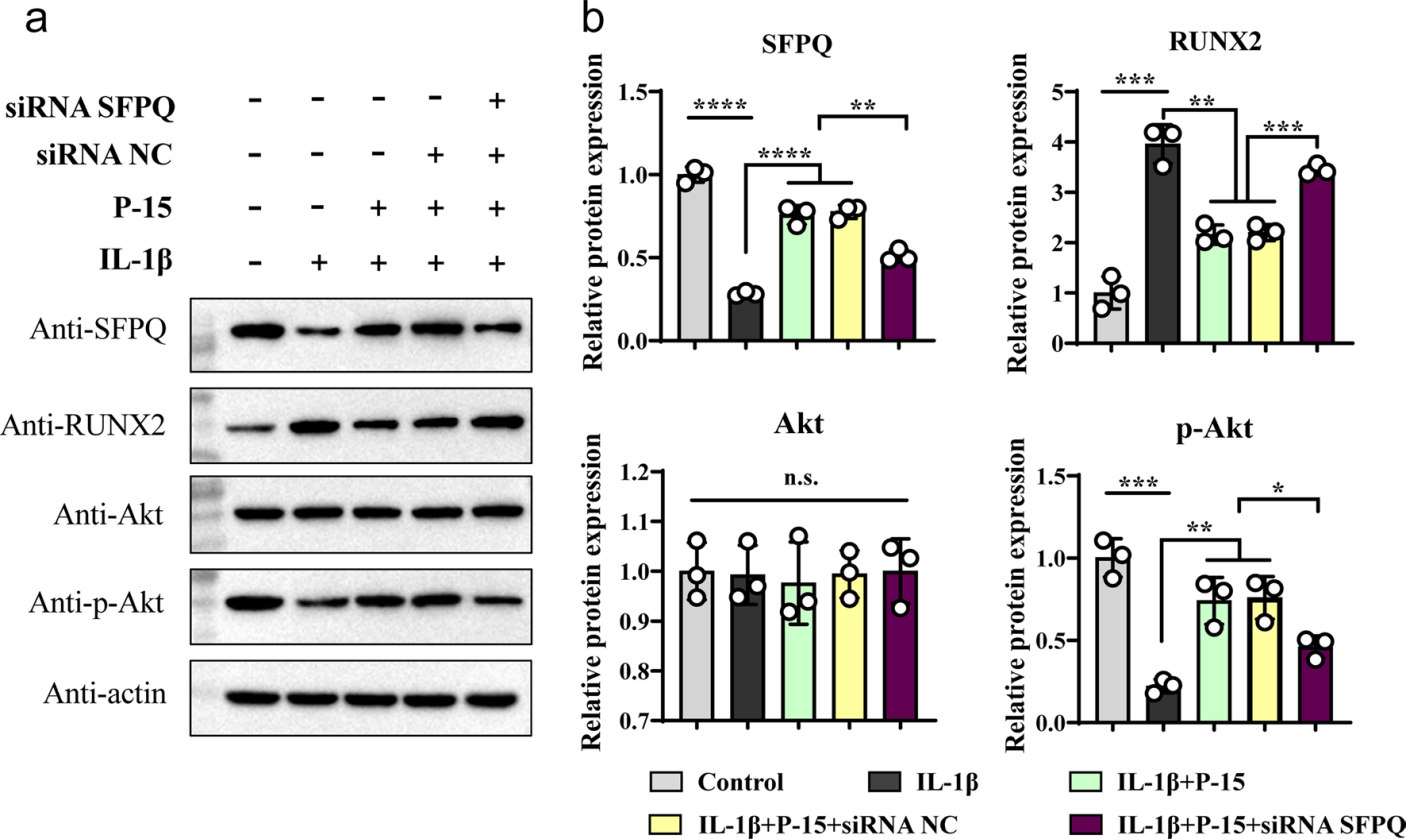
The effect of P-15 on SFPQ/Akt/RUNX2 signaling pathways. **a** Effects of P-15 co-cultured osteoarthritic chondrocytes on pathway proteins SFPQ, Akt, p-Akt, and RUNX2 by Western blot. **b** Quantitative analysis of WB results. Data are presented as mean ± SD. Ns, *P* ≧ 0.05, ^*^P < 0.05, ^**^P < 0.01, ^***^P < 0.001, ^****^P < 0.0001, *n* = 3

Next, we further explored the molecular mechanism of P-15 regulating SFPQ to promote chondrocyte proliferation. Studies have reported that SFPQ could be competitively bound by non-coding RNA, thereby upregulating RUNX2 expression [12]. Therefore, we examined the effect of altered SFPQ expression patterns by P-15 on the Akt-RUNX2 signaling pathway. As shown in Fig. 4, IL-1β significantly inhibited the protein expression of SFPQ, consistent with previous studies, the downregulation of SFPQ promoted the expression of RUNX2 in the opposite direction. Identically, P-15 could reverse the processes induced by IL-1β, as does an inhibitor of IL-1β. In-depth exploration found that IL-1β, P-15, and siRNA SFPQ did not affect the transcription and translation of Akt. But, consistent with the changing trend of SFPQ, IL-1β deeply inhibited the phosphorylation of Akt, which was reversed by P-15. Subsequently, this reversal effect was again reversed by siRNA SFPQ (Fig. 4a and b). Taken together, our data elucidated that P-15 upregulates SFPQ to repress RUNX2 expression, which acts by promoting Akt phosphorylation.

## Discussion

As the only cell type in cartilage tissue, chondrocytes express and secrete a large amount of extracellular matrix (ECM) to maintain the normal function of cartilage [31]. When osteoarthritis occurs, the balance of chondrocyte synthesis and catabolism is disrupted, accelerating the destruction of cartilage [32, 33]. Therefore, protecting chondrocytes from damage is the key to treating osteoarthritis. In the present paper, P-15 treatment significantly increased chondrocyte viability and proliferative capacity, which is important for delaying the development of osteoarthritis. Furthermore, we found that the higher the concentration of P-15, the lower the cell viability, indicating that P-15 has a "dual" effect, which is dependent on the dose used.

SFPQ is involved in multiple nuclear processes, such as pre-mRNA splicing, DNA repair, and transcriptional regulation [34]. It was previously associated with brain dysfunction and telomere instability [35, 36]. Interestingly, lncRNA could bind to SFPQ and elevate the translational levels of RUNX2 through interaction with the IRES domain in the 5’UTR of the corresponding RUNX2 mRNAs by dissociating the SFPQ/polypyrimidine tract-binding protein 2 (PTBP2) dimer [12, 13]. The results suggest that SFPQ participated in the regulation of RUNX2 expression. Moreover, the mechanism of action of RUNX2 is very complex and is a ‘double-edged sword’. On the one hand, RUNX2 is upregulated in a variety of human cancers and is closely associated with tumor metastasis [37, 38]. In knee osteoarthritis cartilage, high expression of RUNX2 and DKK-1 leads to chondrocyte hypertrophy and cartilage extracellular matrix decomposition, promoting cartilage degeneration [14]. On the other hand, RUNX2 is required for proliferation of osteoblastic progenitors [39], regulation of osteoprotegerin expression and is involved in bone formation and bone resorption [40]. Similarly, our study showed that RUNX2 is highly expressed in patients with osteoarthritis, as well as mouse primary chondrocytes stimulated by IL-1β also upregulates RUNX2. In vitro experiments highlight that upregulation of SFPQ expression using P-15 can alter this situation. There is no doubt that SFPQ is a key regulator of the RUNX2 gene, and targeting the SFPQ site may be a potential strategy for the treatment of osteoarthritis, and even various human cancers that presence of high levels of RUNX2 [41].

In addition, SFPQ is also required for paraspeckle formation, i.e., nuclear structures that has been shown to promote survival in multiple myeloma cancer cells [34], and play an extremely important role in mRNA splicing, storage, and processing [42]. We discovered the expression of anabolic genes, including COL2a1, ACAN, and BMP2, were decreased in IL-1β-induced cells, indicating that the reduced anabolic process leads to abnormalities in chondrocytes, which may represent early OA pathology. Then it was highlighted that the expression of SFPQ is associated with the formation of paranuclear plaques. P-15 promotes SFPQ to reverse IL-1β-induced decrease in COL2α1, ACAN and BMP2, and increase in SOX9, COMP. Of course, upregulation of SFPQ levels by P-15 leads to RUNX2 inhibition, and our data is exactly the same. Inconsistently, the transcription factor SOX9 activates collagen II expression and prevents the conversion of proliferating chondrocytes into hypertrophic chondrocytes by directly interacting with RUNX2 and repressing its activity [10, 43]. Our data showed that P-15 reduces IL-1β-induced increase in SOX9, illustrating that in addition to inhibiting RUNX2 by controlling SFPQ access, P-15 might also suppress RUNX2 by inhibiting SOX9 expression. These suggest that P-15 is able to control SOX9 and RUNX2, two antagonistic master transcription factors involved in cell fate determination. Regulation of RUNX2 by P-15 may be a dynamic process involving multiple mechanisms.

Wnt-dependent induction of RUNX2, subsequent binding of RUNX2 to LEF1/TCF proteins [44] may together provide an effective feed-forward loop that sustains expression of osteogenic biomarkers to stimulate osteogenesis [18]. Then, a more in-depth study in this paper elucidated that P-15 regulation of SFPQ promotes the phosphorylation activation of the Akt pathway, thereby changing the expression pattern of RUNX2. Activation of Akt is initiated by recruitment to the plasma membrane through the binding of the PH domain of Akt to the phospholipid PIP3. Subsequently, the T308 site of Akt was phosphorylated by PDK-1, and the S473 site was phosphorylated by MTORC2 [45, 46]. From our results, we guess that P-15 may not be involved in Akt promoter activation and protein degradation processes. Further study needs to reveal the specific mechanism of P-15 involved in Akt protein phosphorylation, including protein interaction, phosphorylation of base sites by kinases, etc.[47]. In addition, the above studies only investigated the effect of P-15 peptide on chondrocytes in vitro. However, the role of P-15 peptide in osteoarthritis needs to be further confirmed in animal models for future studies.

## Conclusion

In summary, we have demonstrated that P-15 peptide can strongly maintain chondrocyte growth *in vitr*o. The osteoarthritis therapeutic effect of p-15 requires SFPQ/Akt/RUNX2 signaling. This novel role of P-15 in osteoarthritis chondrocytes offers an opportunity to develop new strategies for the treatment of osteoarthritis.

## Data Availability

The data used to support the findings of this study are available from the corresponding author.
